# Active versus passive distraction for reducing procedural pain and anxiety in children: a meta-analysis and systematic review

**DOI:** 10.1186/s13052-023-01518-4

**Published:** 2023-08-31

**Authors:** Ting Shen, Xixi Wang, Qiaoyun Xue, Dan Chen

**Affiliations:** Department of Emergency, Children’s Hospital of Nanjing Medical University, No. 72, Guangzhou Road, Gulou District, Nanjing, Jiangsu Province China

**Keywords:** Distraction, Pain, Anxiety, Children, Care, Meta-analysis

## Abstract

**Background:**

Procedural pain is very important in clinical children care. We aimed to evaluate the effects of active versus passive distraction for reducing procedural pain and anxiety in children.

**Methods:**

Two researchers searched the Web of Science, PubMed, EMBASE, Cochrane, SinoMed, Wanfang, China National Knowledge Infrastructure, Weipu databases for the randomized controlled trials(RCTs) on the active versus passive distraction affecting procedural pain and anxiety in children until May 18, 2023. The literature screening and data extraction were carried out by two researchers independently. Review Manager 5.3 software was used for data analysis.

**Results:**

13 RCTs were finally included. 553 children received active distraction intervention and 551 children received passive distraction intervention. There were no significant differences in the children self-reported procedural pain betweent active and passive distraction. The parent-reported procedural pain, medical staff-reported procedural pain, children-reported procedural anxiety, parent-reported procedural anxiety, medical staff-reported procedural anxiety in the active distraction were significant less than that of active distraction. Egger regression analysis showed that there was no publication bias in the results.

**Conclusions:**

Existing evidence suggests that active distraction may be more effective in reducing operational pain and anxiety in children than passive distraction. More studies on the effects of active distraction versus passive distraction in children with larger sample size are needed in the future.

**Supplementary Information:**

The online version contains supplementary material available at 10.1186/s13052-023-01518-4.

## Introduction

Pain is an unpleasant feeling and emotional experience associated with actual or potential tissue damage, or a similar experience. Operational pain is usually related to invasive operation or diagnostic examination such as venipuncture, lumbar puncture and so on. Repeated experience of operational pain can lead to short-term and long-term adverse effects, such as loss of appetite, changes in hormone and metabolic levels, physiological reactions and cognitive behavior changes [1, 2]. More than 50% of hospitalized children and adolescents who received venipuncture experienced moderate to severe pain and anxiety, and these pain-related stresses may affect not only physical, social and cognitive functions, but also emotional and psychological effects on children and their families [3–5]. Although the American Academy of Pediatrics guidelines provide medical staff with advice and techniques for the management of pain control in pediatric patients, pain control in infants and young children is not as good as that in adults [6]. In order to reduce pain, anxiety and fear in children with venipuncture and intravenous catheterization, drug and non-drug treatments are used to control pain in children. Many non-drug treatments have been effectively used to reduce pain in school-age children with adequate cognitive development. one of the most effective non-pharmacological methods is attention distraction, including comics, kaleidoscope, bubble blowing, playing games, virtual reality, etc., which can effectively reduce children’s short-term operational pain [7, 8]. Distraction is based on diverting children’s attention to attracting people and things. A commonly used non-drug pain intervention based on the assumption that children’s ability to deal with pain stimuli is hampered, thereby reducing pain and anxiety, which is used by medical staff and parents to reduce operational pain and anxiety in children [9].

Distraction can be divided into active distraction that requires participants to actively participate in stimulating activities such as playing video games, etc. and passive distraction that does not require children to participate in stimulating interaction such as watching cartoons, listening to music, etc. [10–12]. At present, the relevant systematic review [13] shows that distraction has a significant effect on reducing operational pain in children. Some studies have compared the effects of active distraction and passive distraction on reducing operational pain in children, but the results are different and inconsistent. Therefore, this study aimed to systematically collected and compared the effects of active distraction and passive distraction on reducing operational pain in children, and evaluated the effects of active distraction and passive distraction on reducing operational pain and anxiety in children, to provide evidence support for the care of procedural pain and anxiety in children.

## Methods

This meta-analysis was performed according to the Preferred Reporting Items for Systematic reviews and Meta-Analyses(PRISMA) statement [14].

The two researchers searched the randomized controlled trials(RCTs) on the Web of Science, PubMed, EMBASE, Cochrane, SinoMed, Wanfang, China National Knowledge Infrastructure, Weipu databases about the distraction affecting procedural pain and anxiety in children until May 18, 2023. The keywords of this meta analysis for databases seache as follows: (“complementary” OR “alternative” OR “integrative” OR “nonpharmacologic” OR “active” OR “passive” OR “distraction”) AND (“Venipuncture” OR “blood draw” OR “peripheral cannulation” OR " pain” OR “anxiety” OR “painful procedures” OR “procedural”) AND (“infant” OR “child” OR “adolescent”). Through the combination of subject words and free words, the retrieval strategy was conducted with Boolean operators. At the same time, we sorted out the references of inclusive studies and related topics, in order to obtain the relevant literature as much as possible.

The inclusion criteria of this meta analysis were as follows: (1) study type: randomized controlled trials (RCT); regardless of whether the allocation scheme was hidden or not and the blind method was used. (2) the study population were children aged 1 to 16 years old; (3) active and or passive distractions were used as intervention measures in the process of procedure in children. (4) outcome indicators: the pain and anxiety scale score reported by the children self; medical staff reported pain and anxiety scale score; parents reported pain and anxiety scale score. The exclusion criteria for this meta analysis were as follows: (1) studies on non-drug treatment of cancer and chronic diseases; (2) studies of newborns or patients less than one year old; (4) studies of analgesic intervention in combination with other drugs; and (4) cases, reviews or basic experimental studies.

The literature screening and data extraction were carried out by two researchers strictly according to the inclusion criteria and exclusion criteria, including the author, the year of publication, the consistency of the baseline information, the number of study cases, the nursing measures of the control group and the intervention group, the place of the study, the age of the children, the outcome index and specific values, and the final results were cross-checked. The divergent studies are discussed and determined, and if no agreement can be reached, it is decided by the third researcher.

This meta analysis evaluated the bias risk of the included study according to the Cochrane system Evaluation Manual [15], which requires two researchers to evaluate independently. The evaluation included seven aspects: (1) random allocation method; (2) hidden allocation scheme; (3) whether blind method was applied to research subjects and implementers; (4) whether blind method was applied to medical staff of research results. (5) completeness of outcome data, (6) selective reporting of outcome data, and (7) other sources of bias. The above evaluation work was carried out independently by two researchers, and the divergent studies were discussed and determined, if not determined by the third researcher.

This meta analysis used Review Manager 5.3 software for data analysis. Because different scale evaluation methods are used in each study, recommended by Cochrane Collaborative Network, the standardized mean difference (SMD) wass calculated using 95% confidence interval (CI). All the mean differences shown in the pictures and tables in the result part were SMD. First of all, we analyzed the clinical characteristics and research methodology of the population included in the study in detail, and made a descriptive analysis if there were differences between clinical characteristics and/or research methodology; on the contrary, Cochran Q test and I^2^ were used for quantitative analysis of heterogeneity. If the merged results exist in statistical heterogeneity (P < 0.1), the objects and methodology included in the study should be analyzed again. If there was no specific source of heterogeneity, the random effect model would be used for meta analysis. In the process of merging results, the research data analysis would be removed individually and the fixed effect model would be selected to merge the data again, and the robustness of the results would be tested. On the contrary, the heterogeneity among the studies was small (P ≥ 0.1), and the data were analyzed by fixed effect model for meta analysis. P < 0.05 showed that there was significant difference between the two groups.

## Results

**Fig. 1:**
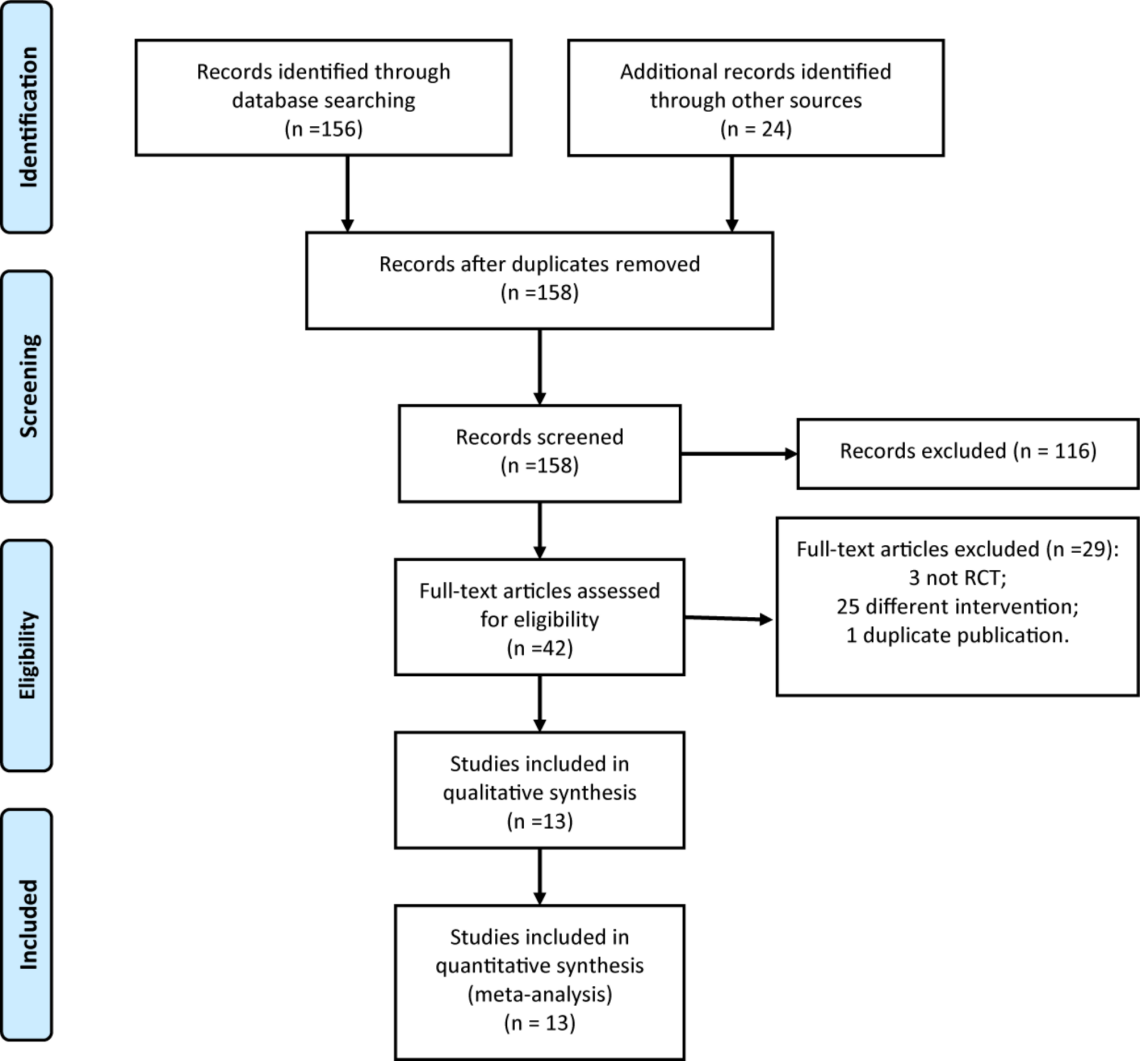
PRISMA flow diagram of RCTs selection

According to the literature retrieval strategy set in this study, a total of 180 articles were retrieved and selected layer by layer according to the inclusion and exclusion criteria, and 13 RCTs [16–28] were included in the final analysis. The flow chart of screening is shown in Fig. 1.

Of the 13 RCTs included, 12 RCTs [16–27] were reported in English and 1 RCT [28] was reported in Chinese. A total of 1104 children were included, 553 children received active distraction intervention and 551 children received passive distraction intervention. Among the 13 RCTs, the types of operational pain included venipuncture, wound dressing change and dental restoration surgery. The research sites included China, Italy, Saudi Arabia, Turkey, Egypt, Sweden and Ireland. The specific features included in RCTs are shown in Table 1.

**Table 1:** Characteristics of included 13 RCTs

RCT	Country	Sample size		Age(years)	Procedure	Intervention	
		Passive distraction	Active distraction			Passive distraction	Active distraction
Abdelmoniem 2016	Egypt	30	30	4 ~ 9	Dental restoration operation	Listen to the same song with headphones	Move the legs up and down as a way to play games
Arıkan 2020	Turkey	72	72	6 ~ 12	Blood sampling collection	Toy wristband	Rotatable wooden toy
Attar 2015	Saudi Arabia	39	39	4 ~ 8	Dental restoration treatment	Local anesthesia and watching TV	Local anesthesia and watching TV through iPad
Aydin 2016	Turkey	30	30	6 ~ 12	Venipuncture	Music of cartoons	Distraction cards: covered with a variety of pictures and shapes, the researchers asked questions about these cards
Aydin 2017	Turkey	50	50	7 ~ 12	Venipuncture	Choose one of the 20 Turkish pop songs stored in the tablet and play it all the way.	Distraction card
Bellieni 2006	Italy	23	23	7 ~ 12	Venipuncture	Watch age-appropriate cartoon movies on TV at least 120 s before venipuncture, and then without other interference.	Mother and child interact and disperse each other by talking, touching, and comforting during venipuncture.
Canbulat 2014	Turkey	62	63	7 ~ 11	Venipuncture	Kaleidoscope	Distraction cards: covered with a variety of pictures and shapes, the researchers asked questions about these cards
Crevatin 2016	Italy	100	100	4 ~ 13	Venipuncture	Nurses instruct children to sing songs, read books, etc.	Tablets play games: angry Birds
Newell 2018	Ireland	24	24	6 ~ 12	Venipuncture	Use the same electronic tablet to watch pre-recorded videos of the same video game	Use the tablet to play games
Nilsson 2013	Sweden	20	20	5 ~ 12	Wound dressing change	Children choose from blue, green, red, orange and yellow lollipops. Lollipops were licked 3–5 min before wound care and lasted the whole course of treatment.	Children began to play 3 ~ 5 min before the start of wound care, and continued to play different game paths throughout the process.
Shekhar 2022	India	41	41	8 ~ 12	Dental treatment	Stress ball	Audio-visual eyeglasses
Xiang 2021	USA	30	31	6 ~ 16	Burn injury wound care	Immersing in the same VR environment without interactions	playing a virtual reality game
Zheng 2011	China	30	30	3 ~ 7	Venipuncture	Showing animated films	Interactive toy

**Fig. 2:**
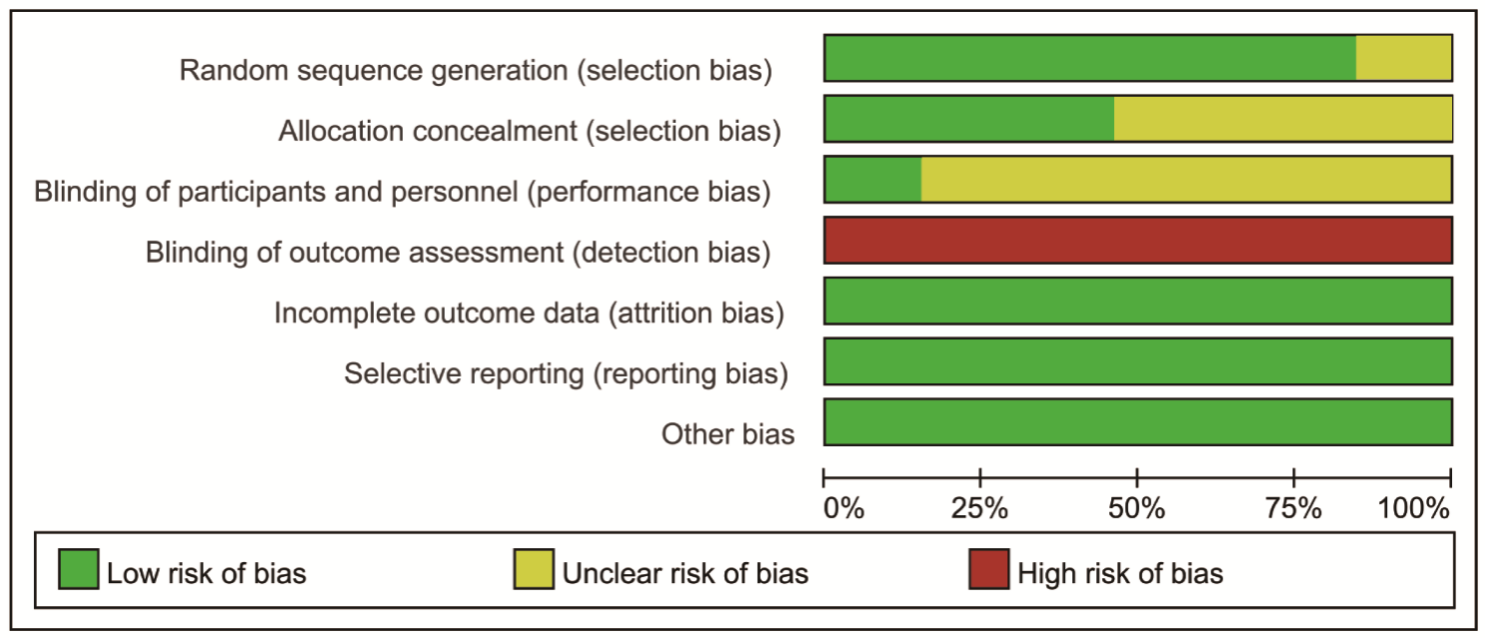
Risk of bias graph

**Fig. 3:**
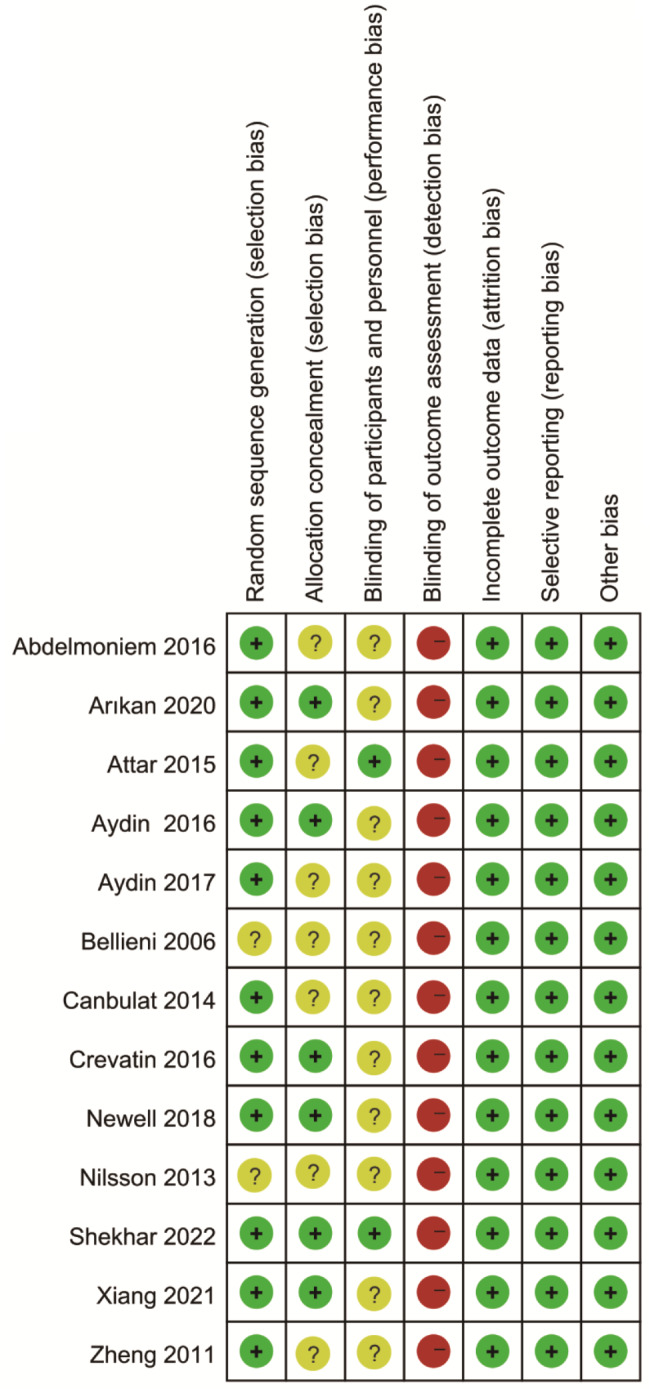
Risk of bias summary

We use the quality evaluation standard of Cochrane manual to evaluate the quality of included RCTs, and the overall literature quality was good. As shown in Fig. 2 and Fig. 3, only 2 RCTs articles did not describe the specific random method. Because of the particularity of the intervention, it was difficult to achieve the blind method of the research object and the intervention, but the blind method of the results evaluator could be used to reduce the bias caused by the blind method of the intervention, but only 2 articles had explained the blind method of the evaluator or the use of two-person independent evaluation to reduce the bias. No other related biases were found amongst the included 13 RCTs.

**Fig. 4:**
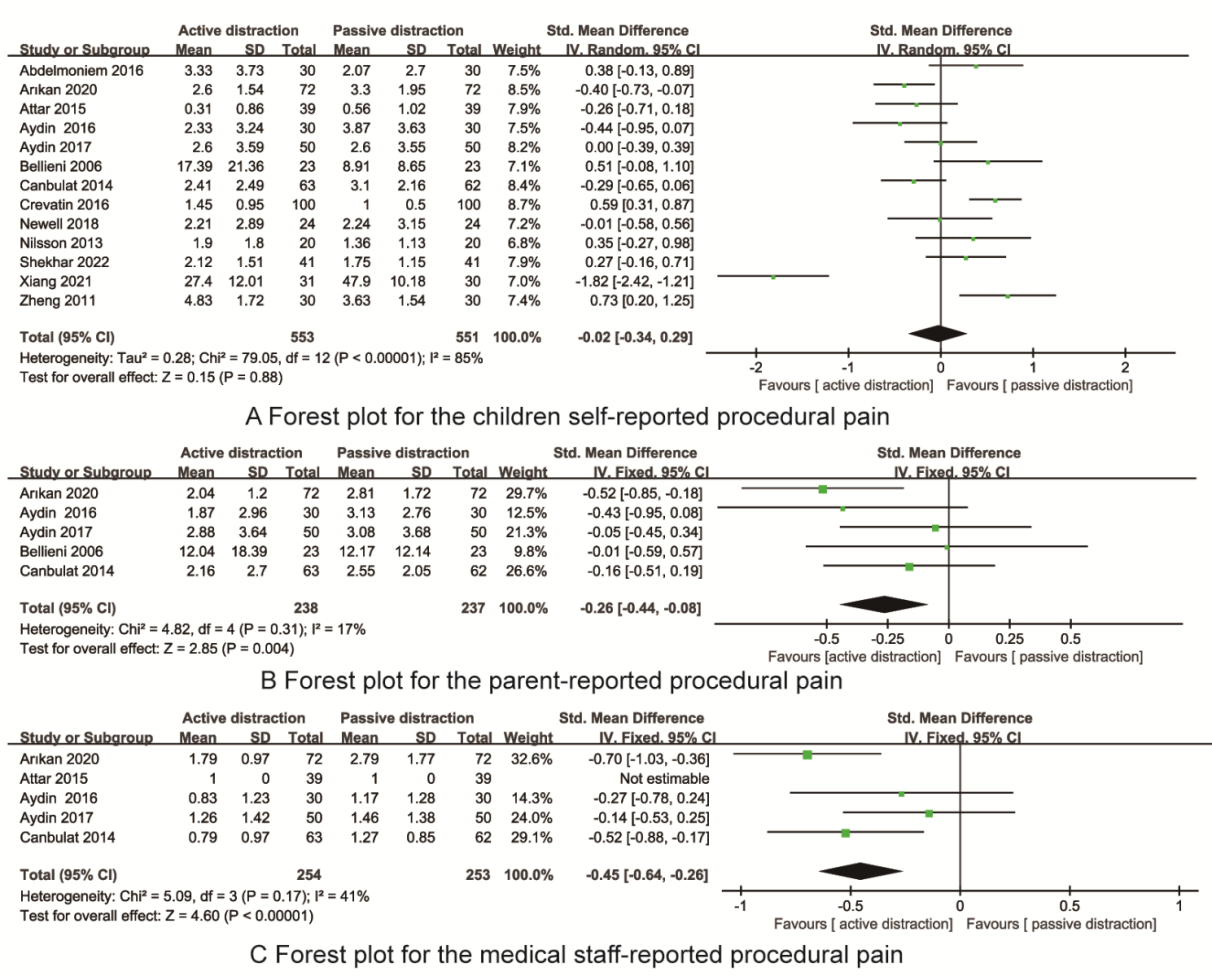
The forest plots for the children self-repo rted, parent-reported and medical staff- reported procedural pain

All 13 RCTs reported the children self-reported procedural pain scores. As shown in Fig. 4A, There were statistically significant heterogneity(I^2^ = 85%, P < 0.01), and random effect model was selected for data analysis. Meta-analysis indicated that there were no significant differences in the children self-reported procedural pain betweent active and passive distraction[SMD=-0.02, 95%CI=(-0.34, 0.29), P = 0.88].

Five RCTs reported the parent-reported procedural pain. As shown in Fig. 4B, There were no statistically significant heterogneity(I^2^ = 17%, P = 0.31), and fixed effect model was selected for data analysis. Meta-analysis indicated that the parent-reported procedural pain in the active distraction was significant less than that of active distraction [SMD=-0.26, 95%CI=(-0.44, -0.08), P = 0.004].

Five RCTs reported the medical staff -reported procedural pain. As shown in Fig. 4C, There were no statistically significant heterogneity(I^2^ = 41%, P = 0.17), and fixed effect model was selected for data analysis. Meta-analysis indicated that the medical staff-reported procedural pain in the active distraction was significant less than that of active distraction [SMD=-0.45, 95%CI=(-0.64, -0.26), P < 0.001].

**Fig. 5:**
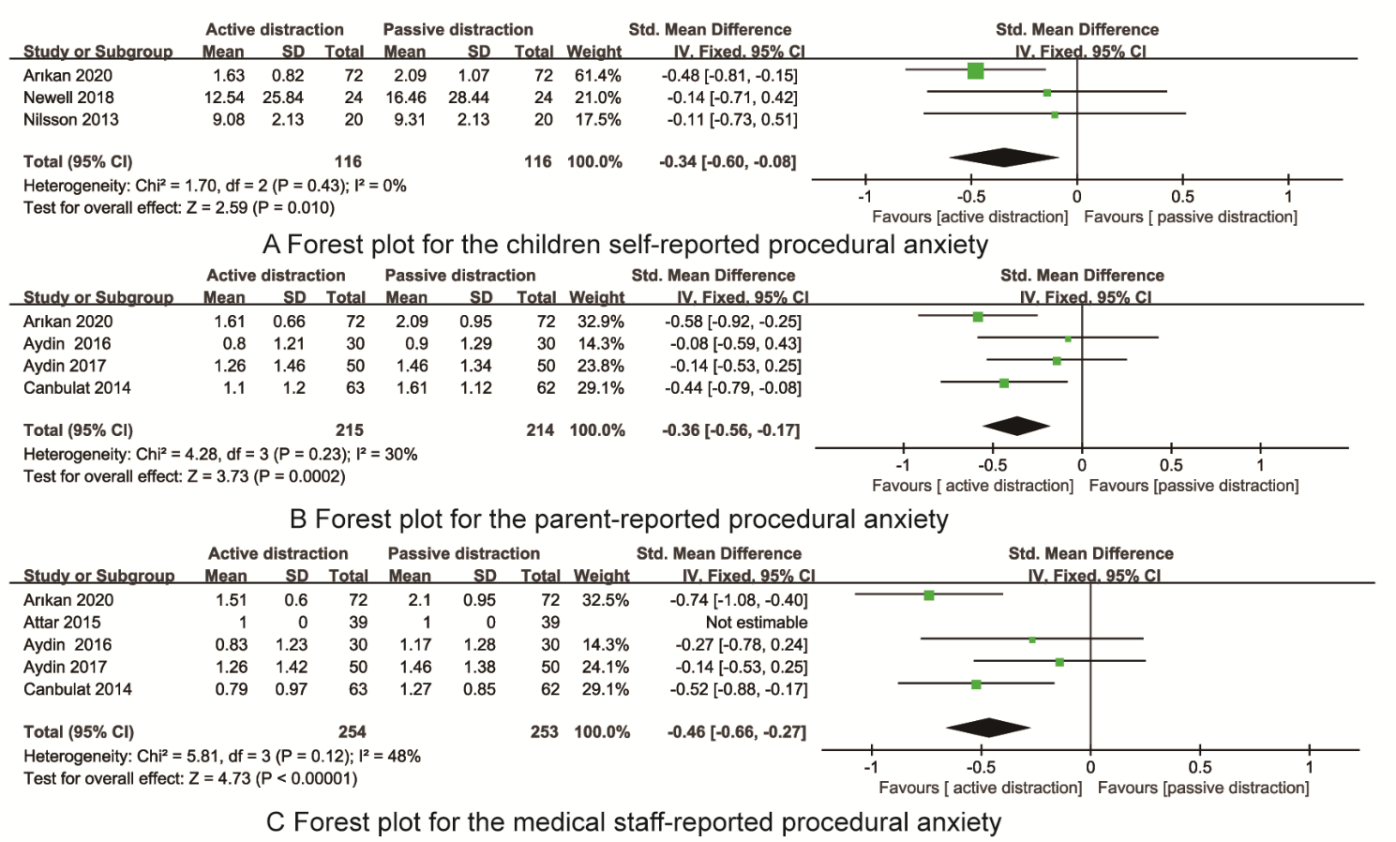
The forest plots for the children self-reported, parent-reported and medical staff- reported procedural anxiety

Three RCTs reported the children-reported procedural anxiety. As shown in Fig. 5A, There were no statistically significant heterogneity(I^2^ = 0%, P = 0.43), and fixed effect model was selected for data analysis. Meta-analysis indicated that the children-reported procedural anxiety in the active distraction was significant less than that of active distraction [SMD=-0.34, 95%CI=(-0.60, -0.08), P = 0.01].

Four RCTs reported the parent-reported procedural anxiety. As shown in Fig. 5B, There were no statistically significant heterogneity(I^2^ = 30%, P = 0.23), and fixed effect model was selected for data analysis. Meta-analysis indicated that the parent-reported procedural anxiety in the active distraction was significant less than that of active distraction [SMD=-0.36, 95%CI=(-0.56, -0.17), P < 0.001].

Five RCTs reported the medical staff -reported procedural anxiety. As shown in Fig. 5C, There were no statistically significant heterogneity(I^2^ = 48%, P = 0.12), and fixed effect model was selected for data analysis. Meta-analysis indicated that the medical staff-reported procedural anxiety in the active distraction was significant less than that of active distraction [SMD=-0.46, 95%CI=(-0.66, -0.27), P < 0.001].

**Fig. 6:**
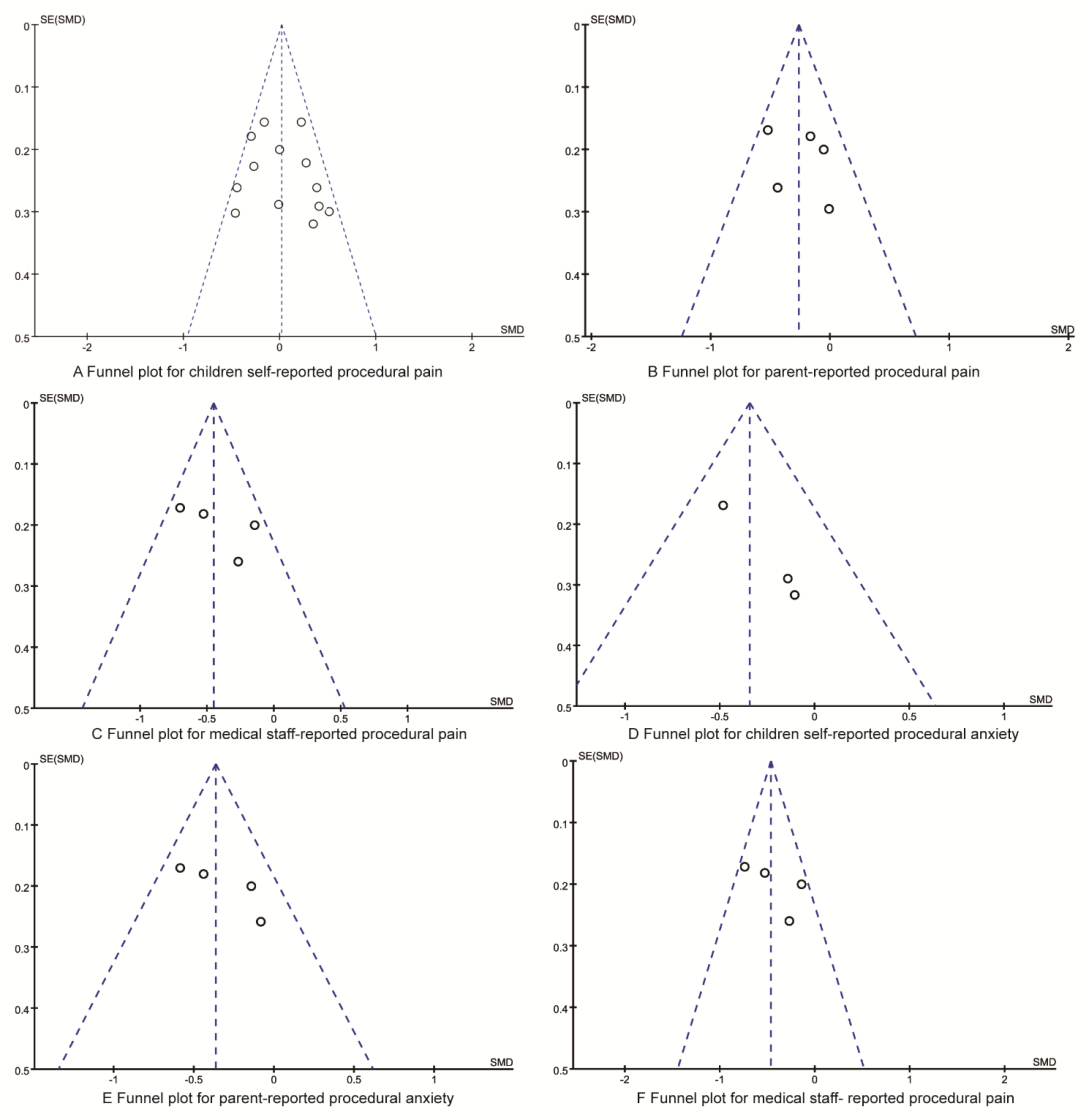
The funnel plots for synthesized outcomes

The results of each synthesied analysis were analyzed by inverted funnel diagram to determine whether there was publication bias. As shown in Fig. 6, the inverted funnel graphs were symmetrical, and the results of Egger regression analysis showed that there was no publication bias in the results (all P > 0.05).

## Discussions

In some cases, drug treatment may cause side effects such as drug allergy, no adverse reactions are found when active or passive distraction interventions are used; and there is no increase in economic cost [29, 30]. And the use of distraction can be implemented through simple training, such as storytelling, watching TV, listening to music, playing with toys or parent interaction, etc., in clinical work, medical staff and parents often distract children during medical operations. However, there is still a lack of evidence-based support in type selection, use time, evaluation effect and intensity [31–33]. Some studies [34, 35] emphasize the importance and necessity for children to choose the type and type of distraction according to their own preferences. Combining the results of 13 RCTs, the meta analysis results of this study show that active distraction can effectively reduce the procedural pain and anxiety of children.

Some studies [36, 37] have shown that distraction can improve children’s cooperation, reduce children’s crying time, reduce children’s plasma cortisol concentration during operation, and reduce children’s discomfort. Previous studies [38, 39] have suggested that children’s pain has not been effectively controlled because the central nervous system of infants and children is considered insufficient to translate, transmit, regulate and perceive pain; due to developments in the field of physiology and behavior, it has been recognized that the central nervous system begins to translate, transmit and regulate nociceptive stimuli from the 23rd week of pregnancy. Studies [40, 41] have confirmed the effectiveness of drug treatment and non-drug intervention in pain, sometimes the use of drugs alone for pain control is not enough, it is recommended to use non-drug treatment in some cases in order to shift the patient’s attention to alternative factors. Distraction is by diverting patients’ attention from medical operations to other things, limiting pain perception, changing operational pain responses and suppressing pain symptoms [42, 43]. The use of distraction techniques is an effective intervention that can improve children’s emotional effects and reduce pain.

Some studies [44, 45] have shown that active distraction has a more significant effect on reducing cold pressor pain, but there are some differences in the clinical environment. The reason for this may be that active distraction requires multi-sensory participation in the interception of pain stimuli, which is generally considered to be better than passive distraction, but for some children who experience pain, it is challenging to participate in active distraction [46]. It mainly depends on the will and ability of the participants. In addition, studies [47, 48] have found that children over the age of 10 benefit from the inclusion of virtual reality technology in video games, while children aged 6 to 10 do not. Because the age span of the children included in this study is large, and the children are not grouped by age, there is no subgroup analysis of age. At the same time, the active distraction methods included in the literature include distraction card, tablet computer, mother-child interaction and so on. The passive distraction methods include watching video, listening to music, lollipop and so on. Some scholars [49, 50] provide customized procedural preparation content through multi-mode distraction devices, which are related to medicine and are suitable for the development of young children, as well as distracting games to immerse children in multi-sensory stimuli. The results show that pain stimulation can be effectively and significantly reduced in the emergency environment. Therefore, it is necessary to implement personalized distraction methods according to children’s age, level of development, temperament and type of treatment and interest [51, 52].

There are some limitations in this study that are worth considering. First of all, part of the research included in this study is that there is a certain heterogeneity between the multiple intervention programs and the passive group. Secondly, the languages of the study are English and Chinese, and the retrieval database is limited, there may be a risk of language bias and incomplete retrieval.

Finally, age can affect the effect of the distraction intervention program. Because there is no effective data in the literature, follow-up study may shorten the age scope of the cihldren or adopt the age-grouped RCT in the future.

## Conclusions

In conclusion, with 13 RCTs included, this meta-analysis has found that active distraction may be more beneficial to reduce the procedural pain and anxiety of children than of passive distraction, but there is still no significnat difference in the children self-reported procedural pain betweent active and passive distraction. Clinically, distraction measures should be reasonably chosen according to children’s age and personal preferences to reduce the procedural pain and anxiety, thereby improving the children experience and care quality.

### Electronic supplementary material

Below is the link to the electronic supplementary material.

Supplementary Material 1

Supplementary Material 2

Supplementary Material 3

## Data Availability

All data generated or analyzed during this study are included in this published article. The original data will be available from corresponding authors on reasonable request.

## List of abbreviations

RCTs: randomized controlled trials
PRISMA: Preferred Reporting Items for Systematic reviews and Meta-Analyses
SMD: standardized mean difference
CI: confidence interval
