# Supplementary material for: Active versus passive distraction for reducing procedural pain and anxiety in children: a meta-analysis and systematic review
Source: Ital J Pediatr. 2023 Aug 31;49:109. doi: 10.1186/s13052-023-01518-4 (PMC10472688; doi:10.1186/s13052-023-01518-4)
Supplement: Supplementary file 2 — Supplementary Material 2 [file 13052_2023_1518_MOESM2_ESM.pdf]

PRISMA 2009 Flow Diagram

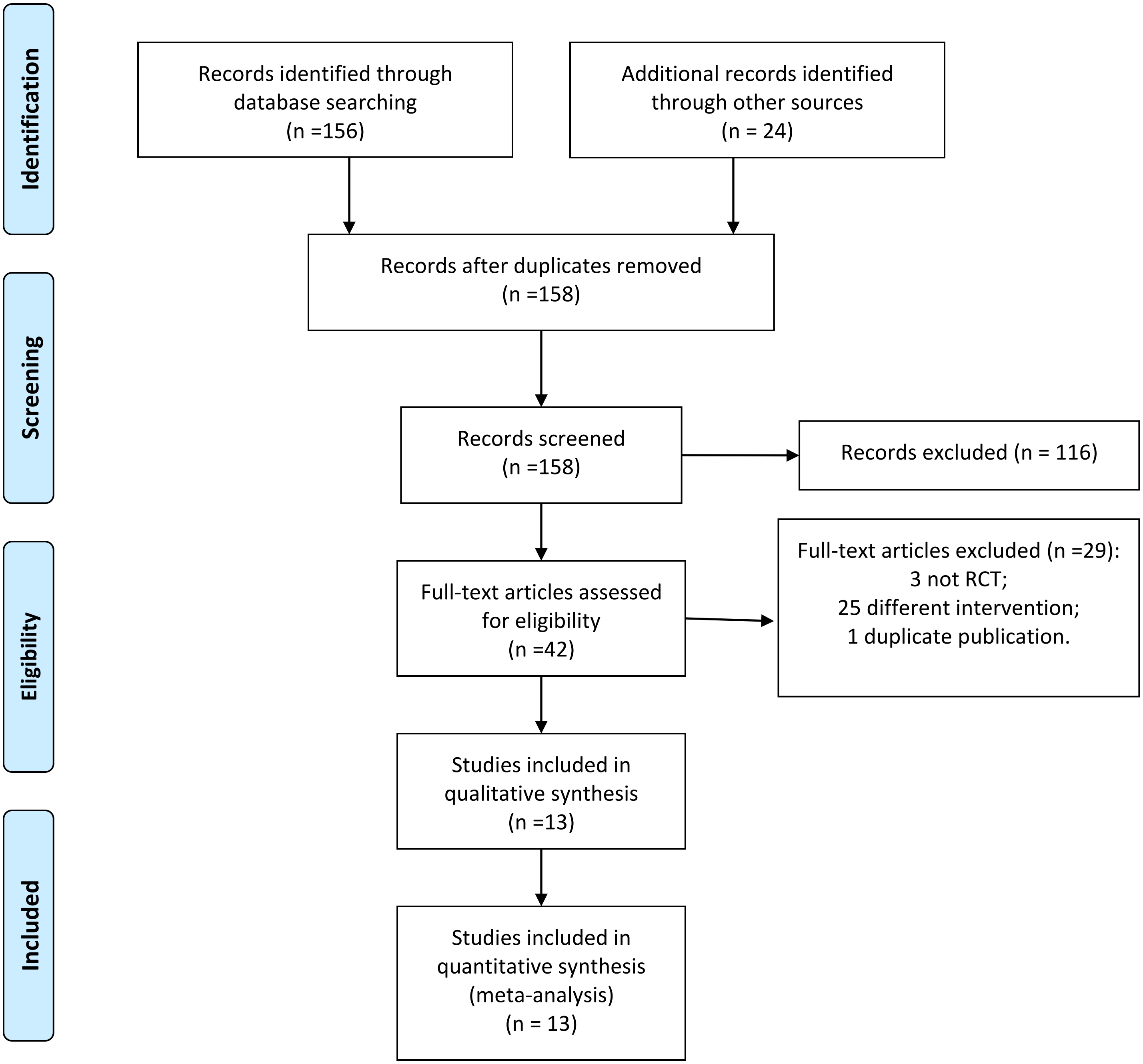

From: Moher D, Liberati A, Tetzlaff J, Altman DG, The PRISMA Group (2009). *P*referred *R*eporting *I*tems for *S*ystematic Reviews and *M*eta-*A*nalyses: The PRISMA Statement. PLoS Med 6(7): e1000097. doi:10.1371/journal.pmed1000097

For more information, visit www.prisma-statement.org.
