# Supplementary material for: Active versus passive distraction for reducing procedural pain and anxiety in children: a meta-analysis and systematic review
Source: Ital J Pediatr. 2023 Aug 31;49:109. doi: 10.1186/s13052-023-01518-4 (PMC10472688; doi:10.1186/s13052-023-01518-4)
Supplement: Supplementary file 3 — Supplementary Material 3 [file 13052_2023_1518_MOESM3_ESM.docx]

Dear editor:

Manuscript ITJP-D-23-00278 entitled “Active versus passive distraction for reducing procedural pain and anxiety in children: a meta-analysis and systematic review", which we submitted to Italian Journal of Pediatric, has now been revised and resubmitted, the responses to the comments of the reviewers are organized one by one at the bottom of this letter.

We sincerely thank you for your tireless patience and reviewer’s wise comments on our manuscript, we marked our responses in red color to distinct from the reviewers’ suggestion, and marked the revised parts in the manuscript with red color, sincerely hope this will simplify your work.

If you have any questions, please do not hesitate to contact us.

Best regards,

Chen

Reviewer reports:

Reviewer #1: hi

It is great meta-analysis article. well organized.

Thank you, we do hope that our study can provide some implication for clinical children care. Thank you.

Try remove all the numbers in the abstract .

Thank you for your kind suggestion, we have removed all the numbers in the abstract as you kindly suggested, please see the revised manuscript.

The suggestions in the attachment file

No need mention to the numbers, those numbers and p=Values are better mentioned in the discussion and the numbers are in the results

Thank you for your kind suggestion, we have removed the numbers and p Values as you kindly suggested, please see the revised abstract section.

where are the terms passive and active ? distraction ???

Thank you, it’s a clerical error, we have added the terms as you discreetly considered, please see the revised method section.

which self-report ?? identify the source of this scale, is the scores are different, what likert scale are those scales ???

Thank you for your discreet consideration, it refers to the self-report pain or anxiety scale reported by the children self, we have explained it as you kindly suggested, please see the revised methods section. The scales are different for different reporters, all the reported scale are well known scale with good reliability and validity.

Don’t write the numbers at the beginning of the sentence

Thank you, we have reivsed the sentence to make it more approperiate, please see the revised manuscript.

Mention to the numbers at the beginning of the sentence as a written like four , five ect..

Thank you, we have reivsed the sentence as you kindly suggested, please see the revised results section.
